# Pharmacokinetics of metal excretion following different doses of sodium EDTA infusion

**DOI:** 10.1093/mtomcs/mfaf010

**Published:** 2025-04-21

**Authors:** Kathrin Schilling, Francisco Ujueta, Siyue Gao, Will A Anderson, Esteban Escolar, Ana Mon, Ana Navas-Acien, Gervasio A Lamas

**Affiliations:** Department of Environmental Health Sciences, Columbia University Mailman School of Public Health, New York, NY, United States; Cardiovascular Medicine Division, Brigham and Women's Hospital, Harvard Medical School, Boston, MA, United States; Department of Environmental Health Sciences, Columbia University Mailman School of Public Health, New York, NY, United States; Department of Environmental Health Sciences, Columbia University Mailman School of Public Health, New York, NY, United States; Columbia University Division of Cardiology, Mount Sinai Medical Center, Miami Beach, FL, United States; Department of Internal Medicine, Mount Sinai Medical Center, Miami Beach, FL, United States; Department of Environmental Health Sciences, Columbia University Mailman School of Public Health, New York, NY, United States; Columbia University Division of Cardiology, Mount Sinai Medical Center, Miami Beach, FL, United States; Department of Internal Medicine, Mount Sinai Medical Center, Miami Beach, FL, United States

## Abstract

Chelation therapy is a promising approach to mitigating health risks associated with toxic metal exposure, which contributes to cardiovascular disease, neurotoxicity, and other chronic conditions. disodium ethylene diamine tetraacetic acid (EDTA) is widely used, but its optimal dosing strategy remains unclear. This study evaluates the dose-dependent efficacy of EDTA in mobilizing toxic metals, including lead (Pb), cadmium (Cd), and gadolinium (Gd), while minimizing the loss of essential metals like copper (Cu) and manganese (Mn) to optimize therapeutic safety and efficacy. Ten volunteers (≥50 years) received 3 infusions at doses of 0.5, 1, and 3 g of EDTA over 30 min, 1 h, and 3 h, respectively. Urine and blood samples were analyzed pre- and post-infusion to assess pharmacokinetics of metal chelation. Urinary Pb excretion increased by 2200% at 0.5 g, with only a marginal gain at higher doses (3300%), supporting low-dose EDTA efficacy. Urinary Cd clearance required 3 g EDTA due to its strong tissue binding. Notably, Gd excretion increased by up to 78 000% even at 0.5 g EDTA, highlighting EDTA's potential to reduce long-term Gd burden post-MRI. Urinary excretion of essential metals varied, with Mn and Zn loss increasing at higher EDTA doses, underscoring the need for dose optimization while Cu and Ca only showed a clear increase urinary excretion at 3 g EDTA. Overall, a 0.5 g EDTA dose effectively mobilized Pb and Gd while minimizing essential metal depletion, reducing infusion time to 30 min, and improving patient compliance. These findings align with TACT and TACT 2 studies, reinforcing EDTA's long-term benefits in Pb reduction and supporting low-dose EDTA as a safe, efficient, and well-tolerated detoxification strategy.

## Graphical Abstract

**Graphical Abstract:**
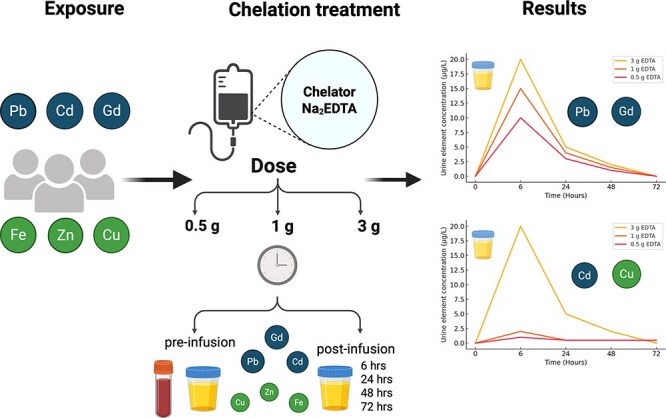

## Introduction

Many trace metals are essential, but some are toxic. As metals play fundamental roles in about 30%–40% of all proteins, synergistic or antagonistic interactions of essential and toxic metal groups determine the risk of diseases for humans. Among xenobiotic metals, cadmium (Cd) and lead (Pb) are of particular concern due to their well-documented toxicity and pervasive environmental presence. Chronic exposure to these metals occurs through inhalation of air pollutants and ingestion of contaminated food and water, leading to bioaccumulation and adverse health effects [1, 2]. Among known toxic chemicals, Pb has been associated with the highest burden of disease, measured in disability-adjusted life years (DALY), exceeding hazardous substances such as per- and polyfluoroalkyl substances (PFAS), phthalates, and polychlorinated biphenyls PCBs [3]. Additionally, both Pb and Cd have been identified by the American Heart Association as cardiovascular risk factors [4], with Pb associated with 250 000 annual cardiac deaths [5].

The significant public health impact of toxic metals is highlighted by their high rankings on the Agency for Toxic Substances and Disease Registry Priority List of Hazardous Substances [6, 7]. While some high-income countries, including the United States, have markedly reduced Pb exposure over the last decades [8] through policies such as the phasing out of leaded gasoline, banning lead-based paints, replacing lead water pipes, and implementing smoking cessation campaigns, low and middle-income countries continue to face substantial challenges in mitigating exposure.

Chelation therapies are effective interventions for reducing the body's toxic metal burden; however, they still remain underutilized. These therapies involve administering chelating agents that bind metals to form stable complexes, which are then excreted via the kidneys or bile. While some chelating agents focus on a single metal, research has shown more promising outcomes for chelating agents that remove multiple metals [9, 10]. disodium ethylene diamine tetraacetic acid (EDTA) is a synthetic chelating agent with a high affinity for divalent metals, including Pb and Cd [11–13]. Although the US Food and Drug Administration (FDA) has approved intravenous use of EDTA for the treatment of Pb poisoning [14], its broader use in treating other diseases or reducing general body metal burden remains controversial [15–19].

Most EDTA chelation trials and small studies used doses of 2–3 g disodium EDTA [9, 17, 20–23], which require prolonged intravenous administration over several hours. This approach carries potential risks such as renal toxicity, particularly in individuals with pre-existing kidney dysfunction [24, 25]. Moreover, EDTA indiscriminately chelates both toxic and essential metals (e.g. Fe and Zn), potentially leading to deficiencies in vital micronutrients. Low-dose EDTA regimens may offer a safer alternative by minimizing adverse effects while maintaining efficacy in toxic metal excretion. However, no comprehensive pharmacokinetic studies have evaluated the optimal dosing regimen of EDTA.

This study aims to optimize EDTA-based chelation therapy by evaluating the excretion profiles of 23 toxic and essential metals and metalloids [antimony (Sb), arsenic (As), barium (Ba), Cd, calcium (Ca), cesium (Cs), chromium (Cr), cobalt (Co), copper (Cu), gadolinium (Gd), iron (Fe), Pb, magnesium (Mg), manganese (Mn), molydbdenum (Mo), nickel (Ni), selenium (Se), strontium (Sr), thallium (Tl), tungsten (W), uranium (U), vanadium (V), zinc (Zn)] following different EDTA dosing regimens. By assessing the effect of dose on metal excretion efficiency, we seek to identify strategies that enhance patient safety, reduce treatment burden, and maximize therapeutic outcomes for specific metals. This study provides critical insights into the potential of tailored chelation therapies to mitigate toxic metal exposure and its associated health risks. Following different doses of EDTA infusions and examined the association with metal excretion to evaluate the effectiveness of chelation therapies.

## Methods

### Study design

The study enrolled 10 volunteers aged 50 years or older, who received three EDTA infusions at weekly intervals. The dosages administered were 500 mg, 1000 mg, and 3000 mg, with each infusion given at a rate of 1000 mg per hour. Urine was collected to measure metal levels at the following time points: preinfusion (*t* = 0), and at 6, 24, 48, and 72 h postinitiation of the intravenous solution. Additionally, blood samples were collected preinfusion. The study was approved by the Mount Sinai Medical Center Institutional Review Board. All participants provided informed consent. Three onsite physicians (G.A.L., E.E., F.U.) were made available during infusions if needed. A trained study coordinator performed a physical examination and obtained vital signs (blood pressure and heart rate) prior to starting an infusion and after infusion administrations. Baseline blood tests were measured at baseline by Quest Diagnostics. These included renal function, serum calcium and albumin, liver function studies, and a complete blood count. To ensure safety, individuals with a baseline creatinine >2 mg/dl were excluded from the trial.

### Inclusion and exclusion criteria

Participant characteristics are summarized in Table 1. Inclusion criteria included male or female >50 years of age, creatinine ≤2 mg/dl, and individuals able to give informed consent. Participants were excluded if aggregate dose >60 g of EDTA was administered within 5 years, allergy to any study drug, blood pressure ≥160/100 mmHg, heart failure hospitalization within 6 months, serum creatinine >2.0 mg/dl, coronary or carotid revascularization within 6 months, tobacco smoking within 3 months and liver disease. All participants stated that they did not receive any MRI in the past 5 years.

**Table 1.** Baseline characteristics of participants

Participant ID	Age (years)	Sex^a^	BUN^a^ (mg/dl)	Serum creatinine (mg/dl)	eGFR^a^ (ml/min/1.73 m^2^)	Comments
1	54	F	20	0.74	97	
2	57	M	14	0.66	111	Stents
3	79	F	16	0.83	72	
4	76	F	15	0.74	84	
5	54	M	16	0.98	92	Stents
6	53	F	12	0.65	106	
7	51	M	19	1.28	68	
8	58	F	15	0.63	103	
9	50	F	10	0.55	112	
10	50	F	15	0.84	85	

a F = female, M = male; BUN = Blood Urea Nitrogen, eGFR = estimated glomerular filtration rate

### Infusions and follow-up

**Figure 1.**
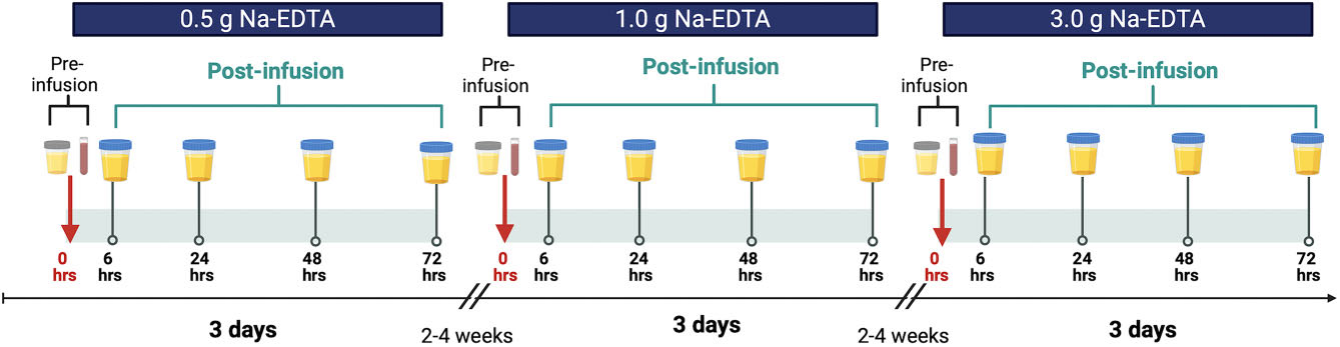
Timeline of chelation study design. Each participant received three infusions: 0.5, 1, and 3 g. Blood and urine samples were collected preinfusion and urine postinfusion after 6, 24, 48, and 72 h.

Details of the EDTA infusions used in this study have been previously described by Lamas *et al*. [26]. The protocol was based on the administration of intravenous EDTA in patients with atherosclerotic disease during the Trial to Assess Chelation Therapy (TACT) [27]. Participants received three infusions over a 2-4-week period, with dosages increasing time (Fig. 1). Infusion 1 consisted of 83 ml of TACT solution containing 0.5 g of EDTA, administered over 30 min. Infusion 2 involved 166 ml of TACT solution with 1 g of EDTA, infused over 1 h. Infusion 3 consisted of 500 ml of TACT solution containing 3 g of EDTA, administered over 3 h. The detailed chemical composition of the infusion is provided in Table S1. A follow-up telephone assessment was conducted 7 ± 2 days after the final infusion to evaluate any delayed infusion reactions.

### Sample preparation for metal analysis

The design of sample collection was based on TACT and TACT2 chelation studies [22, 27]. Whole blood and urine samples were collected using cups, vials, and tubes pretested for metal content and approved for metal analyses. For each infusion, five urine samples and a preinfusion blood sample were collected from each participant (Fig. 1). Preinfusion urine samples were collected as the first morning void on the day of the infusion, and preinfusion blood samples were obtained immediately prior to each infusion.

A time-series approach was used instead of a single 24-h urine collection to better characterize the pharmacokinetics of metal excretion. Four post infusion urine samples were collected at 6, 24, 48, and 72 h after EDTA infusion, using sterile 90 ml containers. Urine collection at multiple time points allows tracking of metal excretion decline, highlighting the shift from rapid to slow clearance. This structured sampling provides a clearer profile of metal mobilization and clearance, yielding more informative data than a pooled urine sample.

Blood samples were collected in three 5 ml tubes immediately prior to the EDTA infusion. Postinfusion blood samples were not collected, as the primary objective of this study was to assess metal elimination, for which urine is a more direct and informative biomarker. While blood metal concentrations can change in response to chelation and may reflect ongoing internal processes such as bone resorption—particularly for metals like Pb [28]—they are also influenced by homeostatic regulation, especially for essential elements. As a result, blood metal levels only may not reliably reflect the amount of metal cleared through chelation over time. Samples were stored at −80°C until analysis.

Trace element analysis of urine and blood samples was conducted at Columbia University's Trace Metal Core Facility using inductively coupled plasma triple quadrupole mass spectrometry (ICP-QQQ-MS). Ultrapure reagents and ultrapure water (≥18.2 MΩ cm) were used for all preparations. Ultrapure nitric acid (HNO_3_, 65%−67%, Optima®) from Fisher Scientific was used for digestion and standard solutions.

Urine samples were prepared by mixing 0.1 ml of urine with 50 µl of an internal standard solution (500 µg/l Ga, Y, Rh, Ir) in 15 ml metal-free polypropylene tubes and diluting to 5 ml with a 2% HNO_3_, 0.02% Triton X-100, and 500 µg/l gold solution. Certified reference materials (CRMs) from Quebec Multi-element External Quality Assessment Scheme (QM-U-Q2022, QM-U-Q2023, QM-U-Q2024), NIST (SRM 2668 Levels 1 and 2), Seronorm L1 (LOT: 1 706 877, Sero, Billing, Norway), and ClinChek Level 1 were used for quality control.

Blood samples were defrosted, homogenized, and 0.1 ml of blood was transferred into 15 ml metal-free tubes, centrifuged at 3000 rpm for 1.45 min. Ultrapure HNO_3_ (0.4 ml) was added, and samples were digested at 80°C for 3 h. After digestion, 40 µl of internal standard solution and 40 µl of gold (50 000 µg/l stock) were added and diluted to 4 ml with ultrapure water. Method blanks were prepared similarly, excluding sample addition. Quality control for blood analysis was performed sing CRMs from Quebec Multi-element External Quality Assessment Scheme (QM-B-Q2003) and Seronorm Blood (LOT: 2 011 933, Sero, Billing, Norway).

### ICP-MS measurement

An Agilent 8900 ICP-MS with an SPS 4 autosampler was used for analysis, equipped with a MicroMist glass nebulizer, glass double-pass spray chamber, Pt/Cu sampler and skimmer cones, and a 2.5 mm quartz plasma torch. Operating conditions included RF power of 1550 W, plasma gas flow at 15.0 l/min, auxiliary gas flow at 0.9 l/min, and a spray chamber temperature of 2°C. External eight-point calibration used matrix-matched solutions.

Nickel (m/z 60), Cu (m/z 65), Sr (m/z 88), Mo (m/z 95), and U (m/z 238), along with internal standards Ga (m/z 69), Y (m/z 89), Rh (m/z 103), and Ir (m/z 193), were measured in helium mode. Mg (m/z 24), Ca (m/z 44), Mn (m/z 55), Co (m/z 59), Zn (m/z 66), Cd (m/z 111), Sb (m/z 121), Cs (m/z 133), Ba (m/z 138), Gd (m/z 157), W (m/z 184), and internal standards Ga, Rh, and Ir were measured in ammonia gas mode. Vanadium (m/z 51→67), Cr (m/z 52), Fe (m/z 56), As (m/z 75→91), Se (m/z 80→96), Tl (m/z 205), Pb (m/z 208), and internal standards Ga, Y, Rh, and Ir were measured in oxygen gas mode.

### Data analysis and quality

Limits of detection (LoD) were calculated as 3.33 × the standard deviation (SD) of blank measurements. Method detection limits (MDL) were derived by multiplying LoD by dilution factors of 50 for urine and 40 for blood. The MDLs for each element anaylzed in urine and blood are provided in Table S1. For quality assurance, all elements determined in the seven certified urine reference materials were found to be within the acceptable range. These results are presented in Table S1.

Urine metal data were normalized for hydration status (see method in Supplemental Information). Urine metals were summarized using means and 95% confidence intervals at baseline (pretreatment) and post-treatment. Comparison of baseline and EDTA treatment with three different doses (0.5, 1, and 3 g) infusions is summarized using percentages for categorical variables and mean and SD. Power analysis was not performed due to the small sample size. All analyses were performed using R software (version 3.6.1).

## Results

### Participants

Ten patients were enrolled with a mean age ± SD of 58 ± 10 years. There were seven (70%) females. All patients had normal renal function during the study enrollment, with a mean (SD) creatinine of 0.79 ± 0.2 mg/dl. No patient had a history of indwelling orthopedic instrumentation. Two patients had a history of coronary artery disease with previous coronary stent placement more than 5 years from study period (Table 1). Two patients had a history of magnetic resonance imaging (MRI) with Gd contrast agent performed for more than 5 years, although no further information on the type of Gd administered was available.

### Toxic metal removal

**Figure 2.**
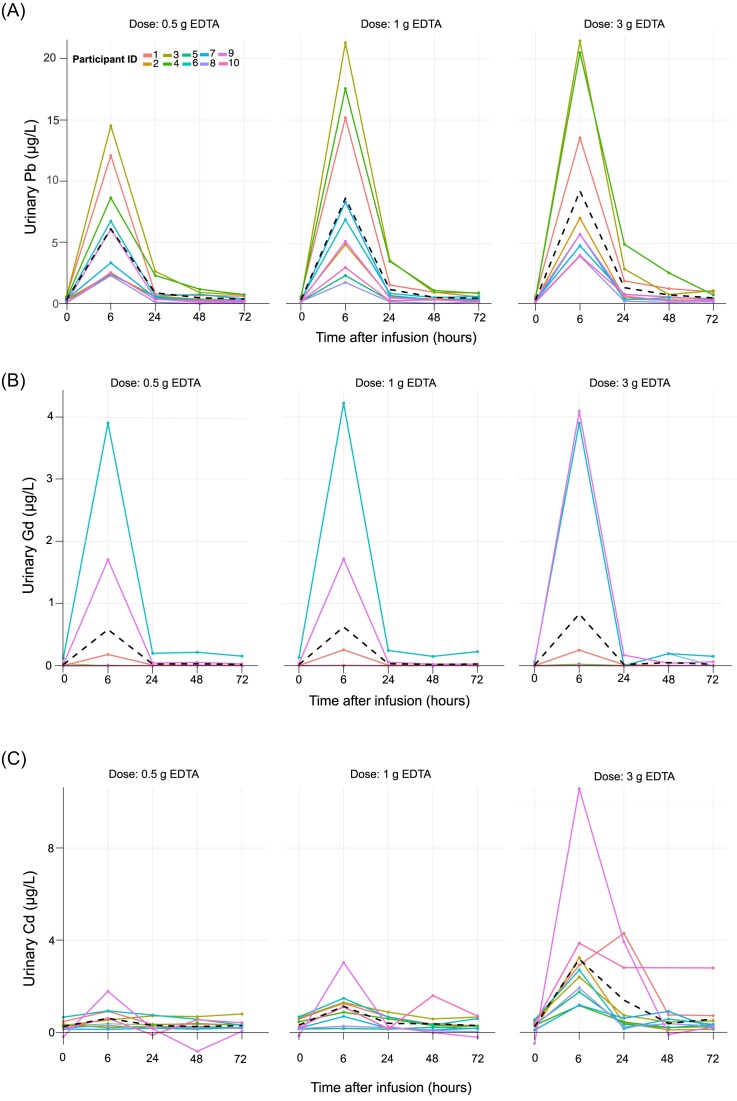
Urinary excretion of toxic metals (A) lead (Pb), (B) gadolinium (Gd), and (C) cadmium (Cd) over time (0, 6, 24, 48, and 72 h) following EDTA infusion at three doses (0.5, 1, and 3 g). Urinary metal concentrations were normalized for hydration status using urine specific gravity.

**Figure 3.**
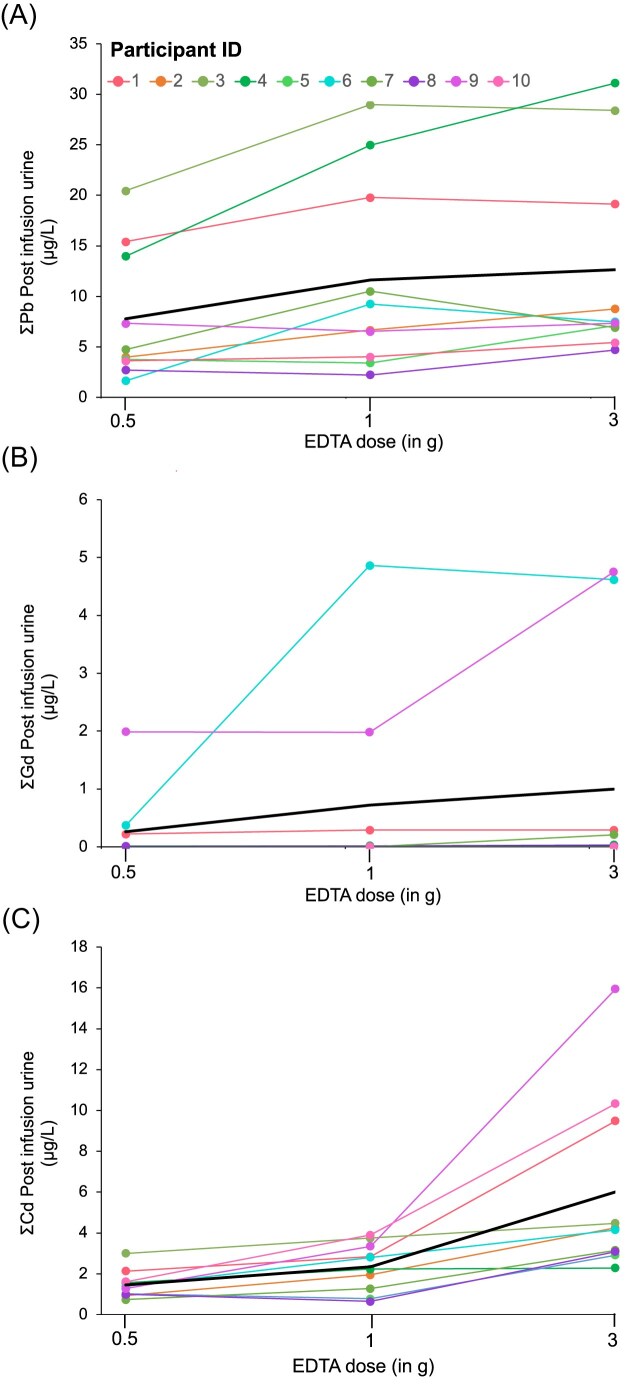
Total urinary excretion of toxic metals—(A) lead (Pb), (B) gadolinium (Gd), and (C) cadmium (Cd)—summed across collection time points (6, 24, 48, and 72 h) for three EDTA infusion doses (0.5, 1, and 3 g) in 10 participants. The black line represents the average excretion of these metals across all participants.

**Figure 4.**
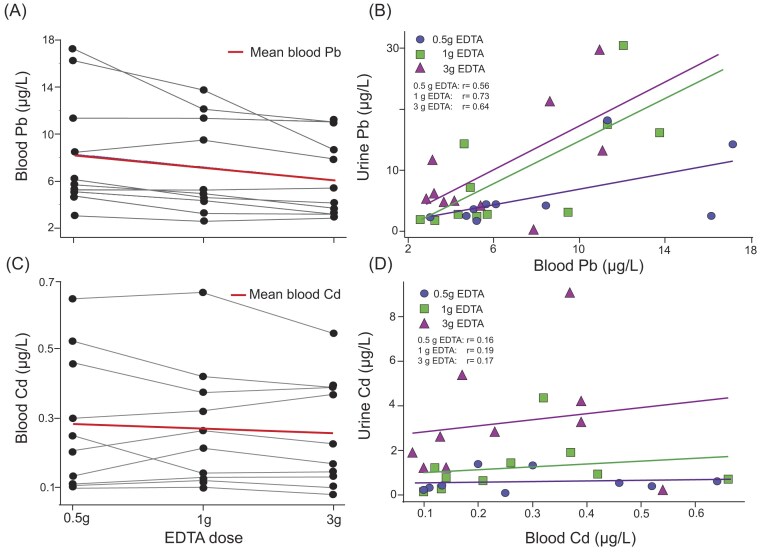
Change in blood levels of (A) lead (Pb) and (C) cadmium (Cd) for 10 participants prior receiving the EDTA infusion of 0.5, 1, and 3 g of EDTA. Association between blood levels of (B) Pb and (D) Cd prior to infusion and urinary clearance after 6 h of infusion for three different EDTA doses.

*Lead (Pb)*: Mean ± SD baseline urinary Pb levels were 0.33 ± 0.18 µg/l across the three infusions and all 10 participants. Urinary Pb levels peaked at 6 h postinfusion at all doses (Fig. 2), with the magnitude of excretion increasing dose-dependently. At 6 h, urinary Pb excretion increased to 7.6 µg/l (95% CI: 4.4–10.9) for the 0.5 g dose, 12.1 µg/l (95% CI: 7.7–16.6) for the 1 g dose, and 12.9 µg/l (95% CI: 7.7–18.0) for the 3 g dose, representing percentage increases of 2200%, 3100%, and 3300%, respectively, relative to baseline. Doubling the dose from 0.5 to 1 g increased Pb excretion by 41%, while a three-fold dose increase from 1 to 3 g yielded only a 4% increase (Fig. 3). Urinary Pb excretion at 6 h was positively correlated with blood Pb levels prior to infusion, with correlation coefficients of *r* = 0.56 (0.5 g), 0.73 (1 g), and 0.64 (3 g) (Fig. 4). Blood Pb levels prior infusions were 8.3 µg/l (95% CI: 4.7–11.9) before the 0.5 g infusion, 7.2 µg/l (95% CI: 4.2–10.1) before the 1 g infusion, and 6.1 µg/l (95% CI: 3.8–8.4) before the 3 g infusion.

*Cadmium (Cd)*: Mean ± SD baseline urinary Cd levels were 0.30 ± 0.28 µg/l. Similar to Pb, urinary Cd levels peaked at 6 h postinfusion across all EDTA doses (Fig. 2). In contrast to Pb, 0.5 g dose produced only a small response for Cd, increasing urinary Cd to 0.67 µg/l (95% CI: 0.23–0.87 µg/l), a 222% increase. This response was more pronounced for the 1 g dose, with excretion rising to 1.24 µg/l (95% CI: 0.38–2.1 µg/l), a 266% increase. The 3 g dose elicited the greatest response, with excretion reaching 3.21 µg/l (95% CI: 1.37–5.04 µg/l), an 820% urinary increase (Fig. 3).

Blood Cd levels prior to infusion showed no significant differences across doses. Mean levels were 0.28 µg/l (95% CI: 0.14–0.42 µg/l) for 0.5 g, 0.27 µg/l (95% CI: 0.14–0.40 µg/l) for 1 g, and 0.25 µg/l (95% CI: 0.14–0.37 µg/l) for 3 g infusions, respectiveley. Correlations between urinary Cd excretion and blood Cd levels were weak, with coefficients of 0.16, 0.19, and 0.17 for 0.5, 1, and 3 g doses, respectively (Fig. 4).

*Gadolinium (Gd)*: Urinary Gd excretion increased significantly from baseline (0 h) to 6 h after EDTA administration in three participants (IDs 1, 6, and 9, see Figs. 2 and 3). The magnitude of excretion varied considerably among individuals, with volunteer 6 showing 4.53 µg/l urinary Gd, far exceeding the average increase of 0.58 µg/l across all participants. On average, the percentage increase in urinary Gd levels for these three participants was 78 000% from baseline to 6 h postinfusion. Importantly, no additional increase in excretion was observed with higher EDTA infusion doses. Blood Gd levels for all participants were below the MDL of 0.009 µg/l in samples collected prior to each infusion.

### Essential metal removal

**Figure 5.**
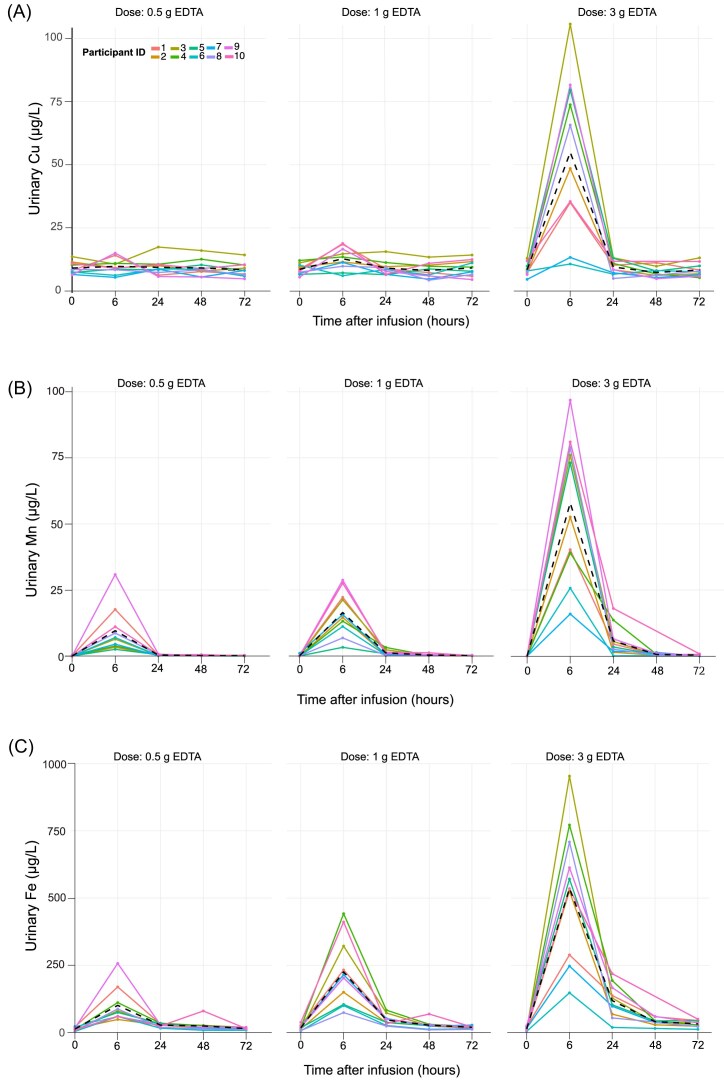

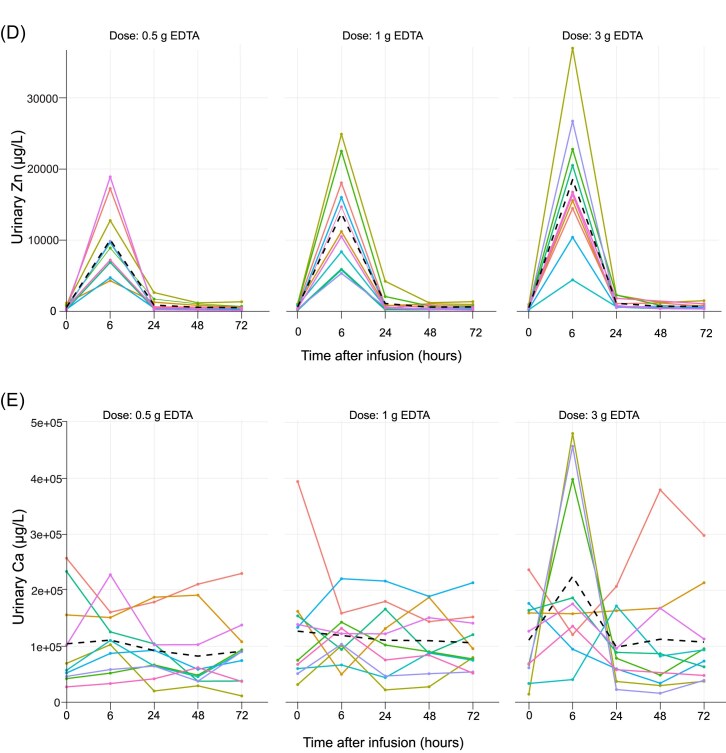
Urinary excretion of essential metals (A) copper (Cu), (B) manganese (Mn), (C) iron (Fe), (D) zinc (Zn), and (E) calcium (Ca) over time (0, 6, 24, 48, and 72 h) following EDTA infusion at three doses (0.5, 1, and 3 g). Urinary metal concentrations were normalized for hydration status using urine specific gravity.

**Figure 6.**
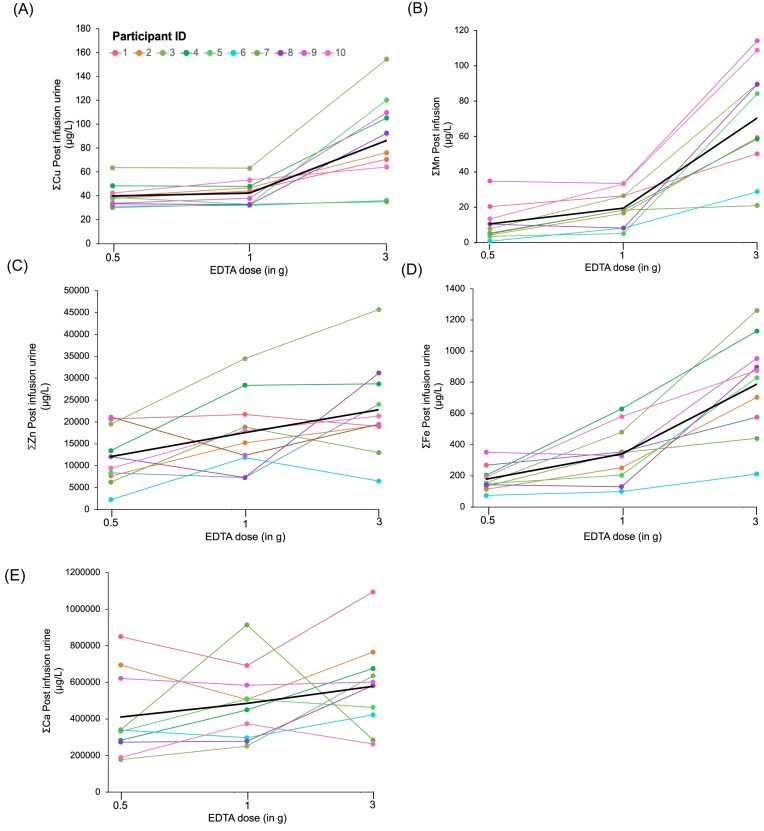
Total urinary excretion of essential metals—(A) copper (Cu), (B) manganese (Mn), (C) iron (Fe), (D) zinc (Zn), and (E) calcium (Ca)—summed across collection time points (6, 24, 48, and 72 h) for three EDTA doses (0.5, 1, and 3 g) in 10 participants. The black line represents the average excretion of these metals across all participants.

*Copper (Cu)*: Mean ± SD baseline urinary Cu levels were 9.5 ± 2.5 µg/l. Urinary Cu excretion peaked at 6 h postinfusion for all doses (Fig. 5). The 0.5 g dose resulted in a small increase to 10.6 µg/l (95% CI: 5.8–13.2 µg/l) of 12% compared to urinary baseline levels. The 1 g dose led to a greater response, increasing excretion to 14.0 µg/l (95% CI: 8.8–19.3 µg/l), an increase of 47% compared to baseline urine. The 3 g dose produced the highest excretion, averaging 64.4 µg/l (95% CI: 34.8–94.1 µg/l), a 678% increase (Fig. 6).

**Figure 7.**
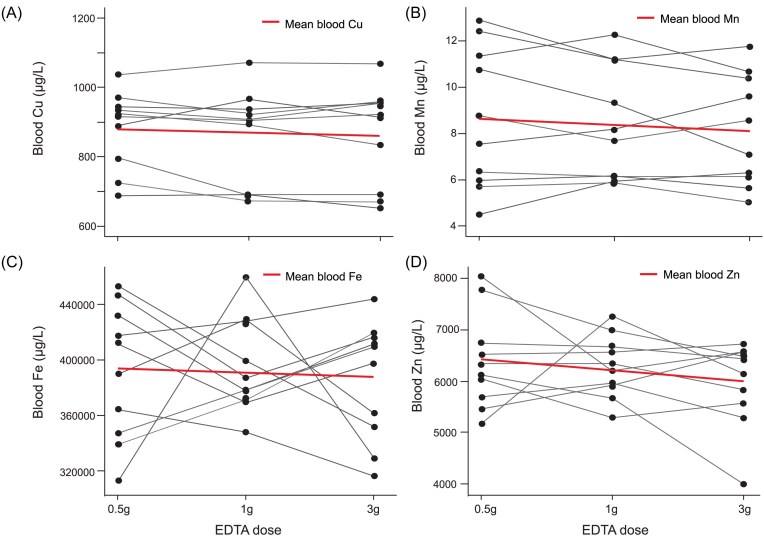
Blood levels of essential metals (A) Cu, (B) Mn, (C) Fe, and (D) Zn for the 10 participants prior receiving the EDTA infusion of 0.5, 1, and 3 g of EDTA. Red line shows the mean blood metal levels for all particpants preinfusion.

Blood Cu levels prior to infusion were consistent across EDTA doses, with means of 882 µg/l (95% CI: 803–961 µg/l) prior to 0.5 g, 865 µg/l (95% CI: 769–962 µg/l) for prior 1 g, and 861 µg/l (95% CI: 759–964 µg/l) prior 3 g infusions (Fig. 7).

*Iron (Fe)*: Mean ± SD baseline urinary Fe levels were 15.9 ± 8.5 µg/l. The 0.5 g EDTA dose increased excretion to 95.4 µg/l (95% CI: 44.6–146 µg/l), a 600% increase compared to baseline. The 1 g dose further increased excretion to 245 µg/l (95% CI: 133–356 µg/l), an increase of 1500% (Figs. 5 and 6). The 3 g dose led to the highest excretion, averaging 626 µg/l (95% CI: 353–898 µg/l), a 3900% rise.

Blood Fe levels showed no significant variation (*P* = .89), with mean values of 392 000 µg/l (95% CI: 357 000–426 000) for 0.5 g, 395 000 µg/l (95% CI: 370 000–419 000) for 1 g, and 386 000 µg/l (95% CI: 355 000–416 000) for 3 g infusions (Fig. 7).

*Zinc (Zn)*: Mean ± SD baseline urinary Zn levels were 524 ± 424 µg/l. The highest Zn excretion occurred consistently at 6 h postinfusion across all doses, indicating a rapid mobilization of Zn stores (Figs. 5 and 6). Variability among participants increased with higher doses, as indicated by the progressively larger SDs. Participants who received the 0.5 g dose exhibited an average Zn excretion of 9600 µg/l (95% CI: 4850–14 360 µg/l). This dose led to an 1800% increase in urinary Zn levels relative to baseline. For the 1 g dose, the average Zn excretion increased to 15 300 µg/l (95% CI: 7650–23 000 µg/l), resulting in a 2500% increase compared to baseline and nearly 40% more than the 0.5 g EDTA dose. The highest dose, 3 g EDTA, led to an average excretion of 21 700 µg/l (95% CI: 11 600–31 800 µg/l) and achieved a 3400% increase, reflecting the most significant response among all three infusion dosages (Fig. 7).

Blood Zn levels remained relatively stable across participants and prior to the three EDTA infusion doses. The mean blood Zn levels were 6384 µg/l (95% CI: 5717–7052 µg/l) for the 0.5 g infusion, 6281 µg/l (95% CI: 5846–6715 µg/l) for the 1 g infusion, and 5956 µg/l (95% CI: 5354–6558 µg/l) for the 3 g infusion. While there was a slight downward trend in mean blood Zn levels with increasing infusion doses, the differences between the infusions were small and fell within the range of the SDs, suggesting no significant change (*P* = .47).

*Manganese (Mn )*: Mean ± SD baseline urinary Mn levels were 0.11 ± 0.21 µg/l. Mn excretion showed a EDTA dose dependent urinary increase, peaking 6 h after infusion (Fig. 5). The 0.5 g dose raised excretion to 9.1 µg/l (95% CI: 3.1–15.2 µg/l), an 8300% increase. The 1 g dose elevated excretion to 18.4 µg/l (95% CI: 9.2–27.6 µg/l), a 17 000% increase, while the 3 g dose further raised it to 64.8 µg/l (95% CI: 37.7–91.8 µg/l), a 58 000% increase (Fig. 6). The blood Mn levels prior to the three infusions showed no significant difference (*P* = .91). The mean blood Mn levels among the participants were 8.63 µg/l (95% CI: 6.4–10.8 µg/l) for 0.5 g EDTA, 8.40 µg/l (95% CI: 6.6–10.2 µg/l) for 1 g EDTA, and 8.11 µg/l (95% CI: 6.4–9.85 µg/l) for the 3 g EDTA infusion, respectively (Fig. 7).

*Calcium (Ca)*: Mean ± SD baseline urine Ca was 124 000 ± 93 000 µg/l with minimal pretreatment variation across doses (Fig. 5). There was a sex difference in baseline urinary Ca levels with 142 000 ± 100 000 µg/l for men and 84 000 ± 41 000 µg/l for women. A clear increase in urinary Ca excretion was observed only in women at 6 hours post-infusion with the 3 g EDTA dose, with levels peaking at 280,000 µg/L—a 107% increase from baseline (Figs. 5 and 6). This clear peak in urinary Ca for a 3 g EDTA dose was not observed for male participants. The mean blood Ca levels among the participants were stable with values of 60 000 µg/l (95% CI: 55 000–65 000 µg/l) for 0.5 g EDTA, 60 000 µg/l (95% CI: 56 000–64 000 µg/l) for 1 g EDTA, and 58 000 µg/l (95% CI: 56 000–62 000 µg/l) for the 3 g EDTA infusion, respectively (Fig. 7).

### Elements with no EDTA chelation response

Elements that did not show a clear dose-dependent response to EDTA, as assessed by urinary excretion, included As, Ba, Co, Cr, Cs, Mg, Mo, Ni, Sb, Se, Sr, Tl, U, V, and W. More information is provided in the supplemental information (Table S1 and Figs S1 and S2).

## Discussion

Our data confirm that EDTA effectively mobilizes and increases urinary excretion of specific metals, with efficacy varying by metal type and dose. The highest urinary excretion levels were observed for the toxic metals Pb, Cd, and Gd, and the essential metals Mn, Fe, Zn, and Cu. For Ca, urinary excretion was only observed for women and for the highest 3 g dose of EDTA. These findings align with previous studies that EDTA is an effective chelator for metals including Pb, Cu, Zn, and Fe [9, 10, 13, 28, 29].

### Mechanism of EDTA-metal chelation

The dose-response relationship for EDTA and urinary metal clearance is nonlinear, reflecting the interaction between metal storage, regulatory mechanisms, and chelation dynamics. The concentrations of 'free' metals in biological systems are very low, typically in the nanomolar to picomolar range, because biological ligands form metal complexes [30]. EDTA's chelating moieties complex divalent metals based on their properties such as ionic charge, radius, and stability constant (log K), with higher stability constants favoring selective chelation. Transition metals generally show high affinity for EDTA due to their charge-to-radius ratio and electronegativity [31, 32]. Among essential metals, Fe^3+^ binds most strongly to EDTA (log *K* = 25.1), followed by Cu^2+^ (log *K* = 18.8), Zn^2+^(log *K* = 16.5), and Mn^2+^ (log *K* = 13.7). For toxic metals, Pb^2+^ (log *K* = 18) and Cd^2+^ (log *K* = 16.9) show strong EDTA binding [9]. Gd^3+^ forms highly stable complexes with EDTA (log *K* = 22) due to its high charge density [33]. EDTA's lower affinity for alkali metals like Ca (log *K* = 10.7) and Mg (log *K* = 8.7) minimizes disruption to electrolyte balance during chelation with EDTA. Metals like Tl^+^ and Mo^6+^ have low EDTA affinity. Our observed differences in metal excretion patterns, beyond the chemophysical properties and EDTA binding affinities, reflect physiological metal storage, protein binding, and regulatory mechanisms that affect chelation kinetics. These findings highlight the need for dose optimization to maximize efficacy while accounting for individual variability.

### Dose-dependent chelation and clinical implications

Most previous EDTA chelation trials and smaller studies have used EDTA doses ranging from 2 to 3 g, typically requiring prolonged intravenous administration over several hours. For instance, Waters *et al*. [9] administered 3 g of EDTA in 14 patients, with only two patients receiving lower doses of 1.2 g and 1.5 g, respectively. Similarly, the TACT and TACT2 trials employed an infusion dose of 3 g [22, 27] and Fulgenzi *et al*. [17] used a 2 g EDTA therapy for the treatment of neurodegenerative diseases. A Cochrane review of five placebo-controlled trials with 1993 participants [18] and a meta-analysis of 24 trials [19] evaluated EDTA chelation. Seventeen studies reported positive health effects, while five found no significant impact.

Our findings demonstrate that a low-dose of 0.5 g EDTA effectively mobilizes Pb, achieving a 2200% increase in urinary Pb excretion from baseline. Although higher doses (e.g. 3 g) resulted in greater absolute excretion (3300% increase), the plateau at 1 g suggests diminishing returns with higher doses (Fig. 3). This plateauing effect highlights the potential of low-dose EDTA to balance effective toxic metal clearance with minimizing adverse effects, such as hypocalcemia. Importantly, urinary Ca excretion only peaked at the 3 g dose and only for female participants, emphasizing the need for dose optimization by sex to avoid adverse effects on Ca homeostasis. The practical advantage of the 0.5 g dose—requiring only 30 min of infusion compared to the 3 h infusion for 3 g EDTA—also reduces patient burden and clinical resource demands. A previous study using a 3 g of EDTA infusion with urine collection immediately afterward showed a 3600% relative increase in Pb levels [10], which is similar to the increase we observed with the same EDTA dose in this study.

The TACT study found that EDTA-based chelation theraphy significantly reduced cardiovascular risk in postmyocardial infarction (MI) patients, with 48% lower risk observed (HR = 0.52, *P* = .0002), especially among those with diabetes [20]. These benefits were attributed to lower blood Pb levels after 40 EDTA infusions, consistent with evidence linking Pb exposure to elevated blood pressure and heart failure [4, 20]. The follow-up TACT2 study, which also focused on post-MI patients with diabetes, was unable to replicate the cardiovascular benefits observed in TACT [22] despite a significantly reduction in the median blood Pb levels after 40 infusions (9.0–3.5 μg/l). Differences in baseline blood Pb levels between the TACT (2003; 14.3 µg/l, NHANES 2003-2004) and TACT2 (2016-2020) populations may explain the differing outcomes [33, 35]. The decline in environmental Pb exposure in the United States over recent decades [2] may have diminished the effectiveness of EDTA treatment during the study period

EDTA does not enter cells and primarily removes metals from circulation. When EDTA reduces the concentration of metals in the bloodstream, the body responds by mobilizing metals from deeper tissue stores and bones to restore equilibrium between reservoirs (=Le Chatelier's Principle). Though EDTA does not act directly on intracellular or bone-stored metals, it creates a concentration gradient that draws them into circulation. Because this process is slow, a single EDTA infusion has little impact on overall metal body burden in adults. Although a recent study suggests that a single EDTA dose can reduce Pb burden in children [28], this effect may not be directly generalizable to older adults. Children typically have lower cumulative Pb exposure and substantially less Pb stored in bone. In contrast, among adults aged 60 and older—our study population—a significant proportion of total body lead burden resides in bone, where it is less readily mobilized. Because EDTA primarily chelates circulating and soft tissue Pb, a single dose may have limited impact on total Pb burden in this older population, where bone turnover is slower and Pb mobilization more gradual. As most toxic metals are not stored in the blood but rather in deeper reservoirs (e.g. Pb in bones and Cd in tissue), repeated infusions are necessary to sustain a continuous gradient that promotes metals to move from tissue stores into circulation. The extended treatment protocol used in TACT and TACT2, which consists of 40 infusions, takes advantage of this gradual redistribution, steadily depleting the body's long-term metal stores, especially of Pb over time.

For Cd, doses of at least 3 g—or extended treatment—are needed to increase mobilization from deeper tissue stores. A 3 g EDTA dose increases Cd excretion by 820% compared to baseline (Figs. 2 and 3). Interestingly, a previous study reported a much lower average increase in urinary Cd excretion (292%) at the same EDTA dose [10], suggesting significant variability in chelation responses across studies. This variability could be attributed to differences in Cd burden, lifestyle factors like smoking, occupational exposures, and genetic predispositions [1, 34, 36]. EDTA efficiently binds Cd during exposure but is less effective once Cd binds intracellularly to metalloproteins like metallothionein [37, 38]. Metallothionein, a cysteine-rich protein, binds Cd due to its chemical similarity to essential metals like Zn and Cu and strong binding affinity [39, 40]. In mice, Cd binds to metallothionein within 5 h of exposure, forming a stable complex (log *K* = 15; [38]). Although Cd-EDTA complexes are even more stable (log *K* = 16.9), EDTA functions only in extracellular spaces, such as blood plasma, because its ionic nature prevents cellular penetration. As a result, intracellular Cd remains inaccessible to EDTA chelation.

EDTA effectively promotes urinary Gd excretion, offering a therapy of lowering Gd body burden after MRI. Urinary Gd excretion increased by 78 000% from baseline for all individual doses >5000% urinary Gd increase. Even a low 0.5 g EDTA dose effectively mobilizes Gd (Figs. 2 and 3) without requiring extensive kidney monitoring, making it a practical and safe post-MRI therapy. Gd-based contrast agents have been integral to enhance MRI image quality and diagnostic accuracy for over three decades [41, 42]. In the United States, millions of Gd-based contrast agents are administered every year [43] but with rising safety concerns. Gd retention in brain tissue and its link to nephrogenic systemic fibrosis in patients with impaired renal function are now well documented [44, 45]. In response to these risks, the US FDA updated its guidelines in 2017 to include warnings about Gd retention in the body. While >95% of Gd-based contrast agents are cleared by the kidneys within 12 h [46, 47], elevated Gd urinary levels have been detected long after MRI [48]. Our study supports this observation, as two participants had urinary Gd baseline levels (0.07 ± 0.05 µg/l), orders of magnitude higher than those of participants who likely never had an MRI (mean baseline urine Gd = 0.002 ± 0.007 µg/l). Both participants had a history of MRI with Gd-based contrast agents more than 5 years prior; however, specific details regarding the type of Gd agent administered were unavailable. These findings suggest Gd can persist in the body in long-term storage such as bones or brain tissue, with slow excretion continuing for over 5 years post-MRI [43–45]. Additionally, blood Gd levels below LoD provide further evidence of Gd mobilization from these storage sites. Our data further demonstrate EDTA successfully chelates Gd even over 5 years post-MRI. Another chelator evaluated for Gd mobilization is 3,4,3-LI(1,2-HOPO), which has been reported to have two orders of magnitude higher Gd affinity than EDTA [42]. However, with 95% of Gd excreted in urine and only 5% in feces, low-dose intravenous EDTA is more effective than oral HOPO chelation. Thus, EDTA is more effective for long-term Gd deposition, while HOPO suits immediate exposure treatment.

A 0.5 g EDTA infusion is a safer alternative for chelation therapy, minimizing the loss of essential metals compared to higher doses (Figs 5 and 6). Prolonged high-dose EDTA treatment (>2 g) depletes essential metals, risking disruption of enzyme activity, oxygen transport, and antioxidant defenses [9, 49–52]. Essential metals, present in concentrations ranging from millimolar to nanomolar levels, compete for EDTA binding. Binding strength to EDTA decreases in the order Fe > Cu > Zn > Mn > Ca, while the abundance of these metals in the body follows the order Ca > Fe > Zn > Cu > Mn. Although Cu has a strong affinity for EDTA (log K Cu > Pb), our data show significant mobilization only at a 3 g dose. At 0.5 and 1 g doses, urinary clearance is negligible compared to baseline. This is likely because Cu is tightly bound to proteins. This aligns with previous studies [9], which found no significant increase in urinary Cu excretion even at a 3 g EDTA dose. Similarly, urinary Mn excretion increased ∼four-fold at the higher 3 g EDTA dose compared to lower doses. The remarkable increase in Mn levels in urine postchelation could be related to a shift in the regular Mn excretion pathway from the biliary tract to the urine [53]. Fe and Zn are chelated even at low doses. However, the risk of depletion is lower with a 0.5 g dose, especially for Zn, where clearance is about half that of a 3 g dose. Monitoring essential metal levels during chelation therapy remains crucial. Nonetheless, the reduced depletion risk makes low-dose EDTA therapy more appealing.

### Comparative global implications

Our study suggests the potential of low-dose EDTA (0.5 g) therapy as a practical and effective strategy to address the toxic metal burden, particularly in low- and middle-income countries (LMICs), where the prevalence of toxic metal exposure remains alarmingly high [54–57]. Globally, the burden of toxic metal exposure remains a critical public health issue. For example, the World Health Organization estimates that Pb exposure accounts for 21.7 million years of DALYs lost annually [3, 58]. While countries like the United States have significantly reduced lead exposure through policy interventions (e.g. an 88% decrease in air lead levels from 2010 to 2022), many countries continue to face rising toxic metal levels [8]. Average blood Pb levels remain alarmingly high in regions like Mali (129 µg/l), Venezuela (72 µg/l), and India (57 µg/l) (https://leadpollution.org/). In such settings, safe, effective, and accessible chelation therapies could play a vital role in mitigating health risks associated with toxic metal exposure, including cardiovascular and respiratory diseases, neurocognitive outcomes, and carcinogenic effects.

High blood Pb levels in children are of special concern as Pb poisoning can affect cognition and neurodevelopment. For instance, in the United States, chelation therapy for Pb poisoning is recommended when a child's blood Pb level is ≥45 µg/dl and the administered dose is usually 1–2 g of EDTA [28, 59, 60]. Our findings demonstrate that a low-dose EDTA regimen is a feasible and effective option for mobilizing and promoting the urinary excretion of Pb. A 0.5 g dose increased urinary Pb excretion by 2200% while preserving essential metals, minimizing systemic effects. The 0.5 g dose, compared to the 3 g dose, is more practical. It requires only 30 min for infusion time, reducing patient burden and clinical resource demands. Given that multiple follow-up chelation visits are commonly employed in occupational medicine and pediatric poisonings, repeated dosing with 0.5 g EDTA may offer a strategic approach to reduce body burden levels after prolonged or high-level exposures. Additionally, the lower dose reduces the quantity of EDTA required per treatment, making it more cost-effective and suitable for deployment in resource-limited settings. Importantly, our findings suggest the lower dose improves safety, reducing risks like hypocalcemia while maintaining efficacy. Even lower dosages (<0.5 g EDTA) could be tested in future, which might be even safer for pediatric use.

### Limitations

This intervention study is limited by its small sample size (*n* = 10) and the nonrandomized design. Nevertheless, it demonstrated the efficacy of 0.5 g EDTA for urinary Pb and Gd clearance from the body. Even lower doses (<0.5 g) were not evaluated, leaving unanswered questions about the minimum effective EDTA dose. Additionally, urinary excretion reflects only a measure of metal clearance, and does not provide information about mobilization of metals from specific tissue reservoirs, such as the liver, kidney, or bone. This is particularly relevant for metals like Cd, which is stored intracellularly and in a strong binding environment. Finally, our study primarily focused on pharmacokinetics of metal excretion and did not evaluate the impact of EDTA treatment on clinical outcomes.

## Conclusion

This study establishes low-dose EDTA chelation therapy as a transformative solution for mobilizing toxic metals such as Pb, Cd, and Gd, combining efficacy, safety, and practicality. Doses under 1 g achieve significant clearance while preserving essential metal homeostasis, with infusion times as short as 30 min—vastly improving feasibility compared to 3-h protocols for 3 g doses. This approach reduces patient burden and is especially suited to resource-limited settings with high toxic metal exposure.

By offering a scalable, cost-effective, and patient-friendly solution, low-dose EDTA therapy can address the global toxic metal burden (especially for Pb), particularly in low and middle-income countries. Tailored regimens and ongoing monitoring can further optimize outcomes. Future research should focus on long-term efficacy, individual variability, and applications in high-exposure areas, cementing low-dose EDTA as a cornerstone of global health strategies against toxic metal-related risks.

## Supplementary Material

mfaf010_Supplemental_File

## Data Availability

Data available on request.
