# Supplementary material for: Pharmacokinetics of metal excretion following different doses of sodium EDTA infusion
Source: Metallomics. 2025 Apr 21;17(5):mfaf010. doi: 10.1093/mtomcs/mfaf010 (PMC12050972; doi:10.1093/mtomcs/mfaf010)
Supplement: mfaf010_Supplemental_File [file mfaf010_supplemental_file.pdf]

# Supplemental information

*for*

**Pharmacokinetics of metal excretion following different doses of ethylenediamine edetate disodium (EDTA) infusion**

Kathrin Schilling PhD[1]*., Francisco Ujueta MD, MS[2]*., Siyue Gao[1], Will Anderson[1], Esteban Escolar MD., Ana Mon MPH., Ana Navas-Acien MD, PhD[1]., Gervasio A. Lamas MD[3,4].

[1] Department of Environmental Health Sciences, Columbia University Mailman School of Public Health, New York, NY

[2] Cardiovascular Medicine Division, Brigham and Women's Hospital, Harvard Medical School, Boston, MA

[3] Columbia University Division of Cardiology, Mount Sinai Medical Center, Miami Beach, FL

[4]Department of Internal Medicine, Mount Sinai Medical Center, Miami Beach, FL

## Method

*Urine Specific Gravity and hydration correction*

Urine specific gravity (SG) was measured using a digital handheld refractometer (ATAGO 4410 PAL-10S) with automatic temperature compensation and a resolution of 0.001. The refractometer was calibrated with deionized water (SG = 1.000), and periodic checks ensured accuracy. For each measurement, 200 µL of urine was placed on the prism, and SG was recorded with ±0.001 precision. Urinary element concentrations (C) were adjusted for hydration status using the Levin-Fahy equation:

$$C = C_{measured} \times (SG_{median} - 1)(SG_{measured} - 1)$$

where $C_{measured}$ is the measured element concentration, $SG_{measured}$ is the urine's specific gravity, and $SG_{median}$ (1.019) represents the median SG for all participants.

## Results

*Quality controls.*

The quality control (QC) and quality assurance (QA) data for urinary metals analysis are summarized in Table S2. LoDs were determined for all measured elements, with values ranging from 0.002 µg/L for U to 86.1 µg/L for Ca. The evaluation of certified urine reference materials confirms that the recovery rates for most trace elements fall within the acceptable range of 80–120%, supporting the accuracy and reliability of the ICP-MS analytical method used in this study. For QMEQAS urine (QM-U-Q2022, QM-U-Q2023, and QM-U-Q1823), recoveries for essential and non-essential elements with only minor deviation for selenium (Se), which exhibited slightly lower recovery (75-88%). The Recipe ClinChek L1 and Seronorm L1 urine CRMs further validated method performance, with recoveries largely within the acceptable range, except for Ba with 122% for Sernorm L1. Similarly, NIST 2668 Level 1 and Level 2 urines aligned well with certified values. The relative standard deviations (RSDs) for most elements were low, confirming the method's precision.

The QC and QA data for blood metals analysis are summarized in the Table S3. LoDs were established for all measured elements, ranging from 0.01 µg/L for Co, Gd, and Tl to 132 µg/L for Zn. The evaluation of certified blood reference materials, Seronorm Blood Level 1 and QMEQAS QM-B-Q2003, demonstrates the accuracy and precision of the analytical methods used in this study. For Seronorm Blood Level 1, recovery rates for most elements fell within or near the acceptable range of 80–120%, with particularly accurate recoveries observed for Fe (108%), Cu (111%), and Zn (107%). However, some elements, such as V (176%) and Cd (142%), exhibited higher recoveries, suggesting potential overestimation for these metals. QMEQAS QM-B-Q2003 showed more consistent recovery rates, generally between 79% and 117%, indicating reliable method accuracy.

*Elements with no EDTA chelation response.*

Unlike the strongly dose-responsive elements such as Mn, Cu, Fe, and Zn, the urinary excretion patterns for these metals remain relatively unchanged regardless of the dose administered. For example, Tl and W were present in very low baseline concentrations, and their urinary levels remained consistently low, with no discernible change post-infusion, even at the highest dose of 3 g EDTA. Magnesium showed high urinary levels, but the values showed no meaningful dose-dependent increase. Other elements, such as Ni, Cr, and Mo showed slight inter-individual variability in baseline levels, but their urinary excretion patterns were largely unaffected by EDTA treatment. Cesium and Sr showed small fluctuations in urinary levels among participants, but the lack of consistent increases with higher doses of EDTA suggests limited chelation efficacy for these elements. For elements like Se and U, baseline excretion levels were moderate but showed negligible variation post-infusion, regardless of the dose. Even at the 3 g EDTA dose, which was expected to elicit the most robust chelation response, no significant changes in urinary levels were observed. Arsenic was uniformly low urinary levels across participants and doses, further confirming that EDTA does not facilitate its excretion.

Table S1: Composition of the EDTA infusion bags which the 10 participants received.

| Pt. ID | 1 | 2 | 3 | 4 | 5 | 6 | 7 | 8 | 9 | 10 |
|---|---|---|---|---|---|---|---|---|---|---|
| Infusion date | 1/31/23 | 2/3/23 | 2/9/23 | 1/26/23 | 1/31/23 | 4/13/23 | 4/14/23 | 6/22/23 | 2/21/23 | 4/6/23 |
| EDTA (0.5 g) | 0.5g | 0.5 g | 0.5 g | 0.5g | 0.5 g | 0.5 g | 0.5 g | 0.5 g | 0.5 g | 0.5 g |
| Procaine | n/a | 16.6 mg | 16.6 mg | n/a | n/a | 16.6 mg | 16.6 mg | 16.6 mg | 16.6 mg | 16.6 mg |
| NaCl 0.9% | 79.7 mL | 78.8 mL | 78.8 mL | 79.7 mL | 79.7 mL | 57.3 mL | 57.3 mL | 57.3 mL | 78.8 mL | 57.3 mL |
| MgCl | n/a | n/a | n/a | n/a | n/a | 0.3 gm | 0.3 gm | 0.3 gm | n/a | 0.3 gm |
| Water (Sterile) | n/a | n/a | n/a | n/a | n/a | 20.8 mL | 20.8 mL | 20.8 mL | n/a | 20.8 mL |
| | | | | | | | | | | |
| Infusion date | 2/21/23 | 2/23/23 | 3/1/23 | 2/10/23 | 2/21/23 | 5/4/23 | 5/5/23 | 7/6/23 | 4/11/23 | 4/18/23 |
| EDTA (1.0 g) | 1g | 1 g | 1 g | 1 g | 1 g | 1 g | 1 g | 1 g | 1 g | 1 g |
| Procaine | 33.2 mg | 33.2 mg | 33.2 mg | 33.2 mg | 33.2 mg | 33.2 mg | 33.2 mg | 33.2 mg | 33.2 mg | 33.2 mg |
| NaCl 0.9% | 157.7 mL | 142.7 mL | 142.7 mL | 157.7 mL | 157.7 mL | 114.5 mL | 114.5 mL | 114.5 mL | 114.5 mL | 114.5 mL |
| Mg Cl | n/a | n/a | n/a | n/a | n/a | 0.7 g | 0.7 g | 0.7 g | 0.7 g | 0.7 g |
| Water (Sterile) | n/a | 15.0 mL | 15.0 mL | n/a | n/a | 41.5 mL | 41.5 mL | 41.5 mL | 41.5 mL | 41.5 mL |
| | | | | | | | | | | |
| Infusion date | 3/7/23 | 4/4/23 | 4/4/23 | 4/4/23 | 3/7/23 | 5/27/23 | 5/27/23 | 7/20/23 | 5/3/23 | 5/4/23 |
| EDTA (3.0 g) | 3g | 3g | 3g | 3g | 3g | n/a | 3 g | 3 g | 3 g | 3 g |
| Procaine | 100 gm | 100 gm | 100 gm | 100 gm | 100 gm | n/a | 100 mg | 100 mg | 100 mg | 100 mg |
| NaCl 0.9% | 345 mL | 345 mL | 345 mL | 345 mL | 345 mL | n/a | 345 mL | 345 mL | 345 mL | 345 mL |
| Mg Cl | 2 g | 2 g | 2 g | 2 g | 2 g | n/a | 2 g | 2 g | 2 g | 2 g |
| Water (Sterile) | 125 mL | 125 mL | 125 mL | 125 mL | 125 mL | n/a | 125 mL | 125 mL | 125 mL | 125 mL |

**Table S1:** Quality performance for urine CRMs from the Quebec Multi-element External Quality Assessment Scheme (QMEQAS), National Institute of Technology (NIST) and ClinChek (RECIPE) and Seronorm (Sero)*

| | QC/QA | Mg | Ca | V | Cr | Mn | Fe | Co | Ni | Cu | Zn | As | Se | Sr | Mo | Cd | Sb | Cs | Ba | Gd | W | Tl | Pb | U |
|---|---|---|---|---|---|---|---|---|---|---|---|---|---|---|---|---|---|---|---|---|---|---|---|---|
| **LoD_Urine (µg/L)** | | 9.86 | 86.1 | 0.038 | 0.271 | 0.100 | 1.543 | 0.008 | 0.113 | 0.341 | 6.711 | 0.096 | 1.086 | 0.295 | 0.070 | 0.010 | 0.011 | 0.006 | 0.118 | 0.006 | 0.018 | 0.051 | 0.251 | 0.002 |
| **QMEQAS QM-U-Q2022** | MEAN (µg/L) *n=11* | 67222 | 118391 | 3.6 | 18.2 | 6.6 | 15.8 | 2.5 | 13.5 | 107.6 | 642.2 | 129.1 | 66.4 | 105.9 | 70.0 | 2.9 | 6.1 | 4.4 | 3.8 | 0.0 | 0.2 | 26.9 | 107.7 | 3.1 |
| | RSD | 2% | 3% | 2% | 2% | 2% | 16% | 2% | 3% | 3% | 3% | 2% | 3% | 1% | 2% | 3% | 1% | 1% | 11% | 226% | 27% | 1% | 15% | 4% |
| | Certified (µg/L) | - | - | 3.8 | 19.2 | 7.3 | - | 2.57 | 13.7 | 107 | 637 | 141 | 88.40 | 114.0 | 68.4 | 3 | 6.42 | 4.51 | 4.18 | - | - | 29.2 | 129 | 3.24 |
| | Recovery | | | 95% | 95% | 90% | | 95% | 99% | 101% | 101% | 92% | 75% | 93% | 102% | 95% | 95% | 97% | 92% | | | 92% | 83% | 96% |
| **QMEQAS QM-U-Q2023** | MEAN (µg/L) *n=10* | 56429 | 151379 | 25.7 | 11.1 | 0.4 | 18.1 | 5.7 | 2.5 | 326.4 | 265.7 | 962.1 | 236.2 | 141.1 | 657.4 | 13.5 | 5.2 | 6.7 | 12.5 | 0.0 | 0.1 | 39.8 | 600.6 | 0.1 |
| | RSD | 3% | 3% | 2% | 2% | 7% | 16% | 1% | 5% | 1% | 3% | 2% | 2% | 1% | 1% | 2% | 1% | 1% | 9% | 141% | 24% | 1% | 13% | 13% |
| | Certified (µg/L) | - | - | 26.8 | 11.3 | 0.529 | - | 5.85 | 2.17 | 338 | 260 | 1040.0 | 268 | 151 | 646 | 14.2 | 5.44 | 6.8 | 13.5 | - | - | 42.2 | 709 | 0.0911 |
| | Recovery | | | 96% | 98% | 83% | | 97% | 117% | 97% | 102% | 93% | 88% | 93% | 102% | 95% | 96% | 99% | 92% | | | 94% | 85% | 103% |
| **QMEQAS QM-U-Q1823** | MEAN (µg/L) *n=9* | 72910 | 164665 | 8.1 | 35.9 | 2.6 | 16.8 | 8.6 | 25.8 | 22.9 | 337.1 | 66.4 | 126.6 | 159.0 | 215.9 | 9.0 | 2.3 | 9.10 | 34.2 | 0.0 | 0.1 | 16.9 | 30.3 | 2.6 |
| | RSD | 3% | 3% | 2% | 2% | 2% | 21% | 1% | 1% | 3% | 5% | 2% | 3% | 1% | 1% | 2% | 1% | 1% | 9% | 287% | 18% | 1% | 15% | 4% |
| | Certified (µg/L) | - | - | 8.46 | 37.4 | 2.87 | - | 8.88 | 26.0 | 22.7 | 337 | 68.83 | 152 | 171 | 213 | 9.57 | 2.38 | 9.25 | 37.1 | - | - | 17.8 | 35.7 | 2.73 |
| | Recovery | | | 96% | 96% | 90% | | 97% | 99% | 101% | 100% | 96% | 83% | 93% | 101% | 94% | 95% | 98% | 92% | | | 95% | 85% | 96% |
| **Recipe Clinchek L1** | MEAN (µg/L) *n=6* | 18742 | 14352 | 19.58 | 3.86 | 3.86 | 44.0 | 1.91 | 3.20 | 58.47 | 213 | 16.11 | 22.68 | 26.38 | 20.2 | 2.27 | 5.9 | 1.11 | 9.54 | 0.00 | 0.02 | 6.78 | 17.8 | 0.01 |
| | RSD | 6% | 3% | 2% | 3% | 6% | 9% | 1% | 5% | 4% | 12% | 2% | 4% | 2% | 3% | 2% | 3% | 2% | 8% | 115% | 63% | 2% | 10% | 44% |
| | Certified (µg/L) | 18600 | - | 21 | 4.08 | 4.09 | 40.6 | 2.05 | 3.25 | 58.2 | 195 | 17 | 29 | - | 20.2 | 2.56 | 6.1 | - | 11 | - | - | 7.38 | 26.4 | - |
| | Recovery | 101% | | 93% | 95% | 94% | 108% | 93% | 98% | 100% | 109% | 95% | 78% | | 100% | 88% | 96% | | 87% | | | 92% | 68% | |
| **Seronorm L1** | MEAN (µg/L) *n=6* | 40086 | 57250 | 0.26 | 7.84 | 0.27 | 8.95 | 0.22 | 1.05 | 25.22 | 291.23 | 88.37 | 9.77 | 69.19 | 17.95 | 0.08 | 2.37 | 5.07 | 4.38 | 0.01 | 0.03 | 0.12 | 1.51 | 0.01 |
| | RSD | 2% | 1% | 6% | 2% | 7% | 6% | 4% | 5% | 2% | 8% | 2% | 4% | 1% | 2% | 14% | 2% | 1% | 14% | 14% | 65% | 22% | 17% | 40% |
| | Certified (µg/L) | 39900 | 64000 | 0.13 | 7.7 | 0.33 | 4.2 | 0.19 | 1 | 26 | 171 | 97 | 10.5 | 74 | 17 | 0.062 | 2.3 | 4.8 | 3.6 | 0.014 | - | 0.106 | 1.51 | 0.017 |
| | Recovery | 100% | 89% | 199% | 102% | 81% | 213% | 118% | 105% | 97% | 170% | 91% | 93% | 94% | 106% | 133% | 103% | 106% | 122% | 101% | | 110% | 100% | 73% |
| **NIST 2668 Level 1** | MEAN (µg/L) *n=4* | 42835 | 74057 | 1.03 | 1.48 | 1.12 | 18.16 | 0.67 | 2.28 | 26.14 | 270.73 | 9.79 | 23.67 | 78.23 | 50.86 | 1.03 | 0.25 | 4.85 | 1.78 | 0.03 | 1.18 | 0.63 | 1.31 | 0.03 |
| | RSD | 4% | 2% | 2% | 25% | 9% | 32% | 2% | 11% | 8% | 30% | 4% | 7% | 2% | 2% | 6% | 15% | 2% | 10% | 37% | 6% | 5% | 21% | 29% |
| | Certified (µg/L) | - | - | 0.98 | 1.08 | 1.08 | - | 0.816 | - | 28.1 | - | 10.81 | - | - | 51.6 | 1.056 | 0.242 | 4.9 | 1.96 | - | 1.252 | 0.719 | 1.234 | 0.034 |
| | Recovery | | | 105% | 137% | 103% | | 82% | | 93% | | 91% | | | 99% | 98% | 102% | 99% | 91% | | 94% | 88% | 106% | 103% |
| **NIST 2668 Level 2** | MEAN (µg/L) *n=4* | 41575 | 71221 | 47.7 | 28.0 | 43.7 | 27.0 | 49.1 | 110.9 | 118.3 | 207 | 195.0 | 21.7 | 75.3 | 1690 | 15.3 | 21.0 | 216 | 231.2 | 0.05 | 58.6 | 99.3 | 110.4 | 11.89 |
| | RSD | 3% | 2% | 3% | 1% | 1% | 30% | 1% | 1% | 1% | 6% | 2% | 3% | 1% | 2% | 1% | 1% | 1% | 6% | 19% | 3% | 1% | 13% | 1% |
| | Certified (µg/L) | - | - | 48.5 | 27.7 | 47.6 | - | 51.8 | 115.3 | 134.1 | - | 213.1 | - | - | 1687 | 16.4 | 22.4 | 221 | 254.6 | - | 62.5 | 115.2 | 137.9 | 13.37 |
| | Recovery | | | 98% | 101% | 92% | | 95% | 96% | 88% | | 92% | | | 100% | 93% | 94% | 98% | 91% | | 94% | 86% | 80% | 89% |

*N= number of preparation and analyses of the certified reference material over the course of the study.

**Table S2:** Quality performance for blood CRMs from the Quebec Multi-element External Quality Assessment Scheme (QMEQAS) and Seronorm (Sero)

| | QC/QA | Ca | Mg | Fe | V | Cr | Mn | Co | Ni | Cu | Zn | As | Se | Sr | Mo | Cd | Sb | Cs | Ba | Gd | W | Hg | Tl | Pb | U |
|---|---|---|---|---|---|---|---|---|---|---|---|---|---|---|---|---|---|---|---|---|---|---|---|---|---|
| | **LoD_Blood (µg/L)** | | | | 0.03 | 1.58 | 0.33 | 0.01 | 0.85 | 2.83 | 132 | 0.16 | 1.39 | 0.49 | 0.17 | 0.03 | 0.18 | 0.02 | 0.32 | 0.01 | 0.14 | 3.31 | 0.01 | 0.48 | 0.004 |
| **Seronorm Blood Level 1** | MEAN (µg/L) *n=9* | 14999 | 18391 | 315953 | 0.35 | 0.77 | 17.5 | 0.16 | 1.24 | 585 | 4983 | 2.66 | 103 | 7.73 | 0.25 | 0.27 | 2.61 | 2.42 | 114 | 0.007 | 0.03 | 1.42 | 0.01 | 9.44 | 0.04 |
| | RSD | 9% | 15% | 7% | 7% | 33% | 4% | 7% | 22% | 3% | 4% | 7% | 4% | 5% | 37% | 10% | 8% | 4% | 3% | 22% | | 33% | 37% | 6% | 30% |
| | Certified | 14200 | 14800 | 31000 | 0.2 | 0.61 | 16.3 | 0.20 | 1.4 | 650 | 4500 | 2.5 | 67 | 7 | 0.28 | 0.29 | 2.3 | 2.3 | 113 | 0.005 | - | 1.63 | 0.009 | 10.3 | 0.04 |
| | Recovery | 106% | 124% | 102% | 176% | 126% | 108% | 80% | 89% | 90% | 111% | 107% | 154% | 110% | 91% | 94% | 113% | 105% | 101% | 142% | | 87% | 62% | 92% | 89% |
| **QMEQAS QM-B-Q2003** | MEAN (µg/L) *n=6* | - | - | - | 1.85 | 2.38 | 37.5 | 0.34 | 10.1 | 1008 | 6614 | 22.44 | 314 | 14.5 | 4.23 | 1.47 | 8.18 | 3.82 | 7.93 | 0.00 | -0.02 | 12.9 | 4.06 | 163 | 0.78 |
| | RSD | | | | 1% | 23% | 1% | 5% | 20% | 2% | 5% | 2% | 1% | 2% | 3% | 4% | 3% | 3% | 4% | | | 5% | 1% | 1% | 12% |
| | Certified | - | - | - | 1.95 | 2.5 | 39.2 | 0.43 | 10.3 | 1110 | 5960 | 19.1 | 169 | 15.1 | 4.48 | 1.54 | 7.01 | 3.84 | 8.27 | - | - | 12 | 4.55 | 188 | 0.85 |
| | Recovery | | | | 95% | 95% | 96% | 79% | 98% | 91% | 111% | 117% | 186% | 96% | 94% | 96% | 117% | 99% | 96% | | | 108% | 89% | 87% | 92% |

**Table S3:** Change in urinary levels after different dosage of EDTA infusion.

| Element | Time (hours) | EDTA = 0.5g | EDTA = 1g | EDTA = 3g |
|---|---|---|---|---|
| **Arsenic (As)** | 0 | 1.00 (Reference) | 1.00 (Reference) | 1.00 (Reference) |
| | 6 | 1.09 (0.73, 1.61) | 1.79 (1.06, 3.02) | 0.93 (0.69, 1.25) |
| | 24 | 1.20 (0.81, 1.78) | 1.28 (0.76, 2.16) | 0.90 (0.67, 1.21) |
| | 48 | 1.51 (1.02, 2.23) | 1.21 (0.72, 2.05) | 0.67 (0.49, 0.91) |
| | 72 | 1.55 (1.04, 2.29) | 1.64 (0.97, 2.76) | 0.77 (0.57, 1.04) |
| **Barium (Ba)** | 0 | 1.00 (Reference) | 1.00 (Reference) | 1.00 (Reference) |
| | 6 | 0.81 (0.55, 1.20) | 0.83 (0.61, 1.14) | 0.90 (0.57, 1.42) |
| | 24 | 1.02 (0.69, 1.50) | 0.93 (0.68, 1.27) | 1.23 (0.78, 1.93) |
| | 48 | 0.73 (0.49, 1.07) | 0.90 (0.66, 1.23) | 1.06 (0.67, 1.69) |
| | 72 | 0.73 (0.50, 1.08) | 0.99 (0.73, 1.36) | 1.18 (0.75, 1.86) |
| **Calcium (Ca)** | 0 | 1.00 (Reference) | 1.00 (Reference) | 1.00 (Reference) |
| | 6 | 1.21 (0.79, 1.84) | 1.11 (0.78, 1.59) | 2.08 (1.12, 3.86) |
| | 24 | 0.97 (0.64, 1.48) | 0.91 (0.64, 1.30) | 0.95 (0.51, 1.76) |
| | 48 | 0.82 (0.54, 1.24) | 0.96 (0.68, 1.37) | 0.84 (0.45, 1.60) |
| | 72 | 0.88 (0.58, 1.35) | 0.97 (0.68, 1.38) | 1.01 (0.54, 1.87) |
| **Cadmium (Cd)** | 0 | 1.00 (Reference) | 1.00 (Reference) | 1.00 (Reference) |
| | 6 | 1.73 (1.10, 2.72) | 2.66 (1.61, 4.38) | 8.20 (4.38, 15.38) |
| | 24 | 1.13 (0.71, 1.80) | 0.96 (0.58, 1.58) | 2.41 (1.29, 4.52) |
| | 48 | 1.21 (0.76, 1.91) | 0.88 (0.53, 1.47) | 1.38 (0.71, 2.67) |
| | 72 | 0.89 (0.57, 1.40) | 0.84 (0.50, 1.40) | 1.19 (0.63, 2.22) |
| **Cobalt (Co)** | 0 | 1.00 (Reference) | 1.00 (Reference) | 1.00 (Reference) |
| | 6 | 1.12 (0.88, 1.44) | 1.39 (1.07, 1.80) | 2.38 (1.73, 3.27) |
| | 24 | 1.16 (0.91, 1.49) | 1.07 (0.82, 1.39) | 1.21 (0.88, 1.66) |
| | 48 | 1.13 (0.88, 1.45) | 1.08 (0.83, 1.41) | 1.09 (0.79, 1.52) |
| | 72 | 0.99 (0.77, 1.27) | 1.14 (0.88, 1.48) | 1.29 (0.94, 1.77) |
| **Chromium (Cr)** | 0 | 1.00 (Reference) | 1.00 (Reference) | 1.00 (Reference) |
| | 6 | 0.78 (0.44, 1.36) | 1.25 (0.67, 2.34) | 1.31 (0.94, 1.83) |
| | 24 | 0.90 (0.51, 1.57) | 1.64 (0.89, 3.03) | 1.05 (0.75, 1.46) |
| | 48 | 0.98 (0.56, 1.72) | 1.04 (0.56, 1.94) | 0.90 (0.64, 1.28) |
| | 72 | 0.92 (0.52, 1.61) | 0.99 (0.54, 1.83) | 0.80 (0.56, 1.13) |
| **Cesium (Cs)** | 0 | 1.00 (Reference) | 1.00 (Reference) | 1.00 (Reference) |
| | 6 | 0.99 (0.82, 1.20) | 0.97 (0.82, 1.14) | 1.07 (0.87, 1.32) |
| | 24 | 1.04 (0.87, 1.26) | 0.95 (0.81, 1.12) | 0.81 (0.66, 1.00) |
| | 48 | 1.10 (0.91, 1.33) | 0.92 (0.78, 1.09) | 0.77 (0.62, 0.95) |
| | 72 | 1.06 (0.88, 1.27) | 0.98 (0.83, 1.16) | 0.86 (0.69, 1.05) |
| **Copper (Cu)** | 0 | 1.00 (Reference) | 1.00 (Reference) | 1.00 (Reference) |

| | | | | |
|---|---|---|---|---|
| | 6 | 1.06 (0.86, 1.30) | 1.44 (1.13, 1.83) | 5.44 (3.93, 7.54) |
| | 24 | 1.02 (0.83, 1.24) | 1.04 (0.82, 1.33) | 1.12 (0.81, 1.55) |
| | 48 | 0.99 (0.81, 1.21) | 0.92 (0.72, 1.17) | 0.89 (0.64, 1.25) |
| | 72 | 0.91 (0.74, 1.12) | 1.03 (0.81, 1.31) | 0.98 (0.71, 1.36) |
| **Iron (Fe)** | 0 | 1.00 (Reference) | 1.00 (Reference) | 1.00 (Reference) |
| | 6 | 7.88 (5.64, 11.01) | 12.88 (9.59, 17.30) | 39.12 (28.55, 53.61) |
| | 24 | 2.38 (1.70, 3.32) | 2.89 (2.15, 3.88) | 8.18 (5.97, 11.20) |
| | 48 | 1.78 (1.28, 2.49) | 1.60 (1.19, 2.15) | 3.22 (2.33, 4.46) |
| | 72 | 1.26 (0.90, 1.76) | 1.27 (0.94, 1.70) | 2.45 (1.79, 3.36) |
| **Gadolinium (Gd)** | 0 | 1.00 (Reference) | 1.00 (Reference) | 1.00 (Reference) |
| | 6 | 6.60 (1.40, 31.17) | 12.86 (7.78, 21.27) | 10.21 (2.76, 37.86) |
| | 24 | 1.27 (0.23, 6.94) | 1.87 (1.11, 3.16) | 2.74 (0.60, 12.51) |
| | 48 | 2.20 (0.47, 10.36) | 1.33 (0.80, 2.20) | 2.92 (0.69, 12.34) |
| | 72 | 1.04 (0.21, 5.05) | 2.16 (1.27, 3.68) | 1.32 (0.32, 5.39) |
| **Magnesium (Mg)** | 0 | 1.00 (Reference) | 1.00 (Reference) | 1.00 (Reference) |
| | 6 | 0.83 (0.63, 1.09) | 0.86 (0.61, 1.23) | 1.96 (1.39, 2.76) |
| | 24 | 1.17 (0.89, 1.54) | 1.25 (0.88, 1.78) | 1.89 (1.34, 2.66) |
| | 48 | 0.92 (0.70, 1.21) | 0.93 (0.65, 1.33) | 1.44 (1.01, 2.05) |
| | 72 | 0.90 (0.69, 1.19) | 0.99 (0.70, 1.41) | 1.09 (0.78, 1.54) |
| **Manganese (Mn)** | 0 | 1.00 (Reference) | 1.00 (Reference) | 1.00 (Reference) |
| | 6 | 137 (70.52, 264) | 197 (97.20, 397) | 898 (457, 1763) |
| | 24 | 6.92 (3.58, 13.40) | 12.25 (6.06, 24.77) | 55.66 (28.35, 109.28) |
| | 48 | 2.81 (1.45, 5.43) | 4.79 (2.37, 9.68) | 9.57 (4.77, 19.18) |
| | 72 | 2.08 (1.08, 4.03) | 2.48 (1.23, 5.02) | 5.36 (2.73, 10.53) |
| **Molybdenum (Mo)** | 0 | 1.00 (Reference) | 1.00 (Reference) | 1.00 (Reference) |
| | 6 | 0.82 (0.54, 1.26) | 0.82 (0.50, 1.33) | 1.30 (0.87, 1.95) |
| | 24 | 1.11 (0.72, 1.70) | 0.93 (0.57, 1.51) | 0.93 (0.62, 1.39) |
| | 48 | 1.19 (0.78, 1.83) | 0.71 (0.44, 1.16) | 0.70 (0.46, 1.06) |
| | 72 | 0.91 (0.60, 1.40) | 0.75 (0.46, 1.22) | 0.93 (0.62, 1.39) |
| **Nickel (Ni)** | 0 | 1.00 (Reference) | 1.00 (Reference) | 1.00 (Reference) |
| | 6 | 1.28 (0.72, 2.28) | 1.54 (1.05, 2.24) | 1.91 (1.31, 2.78) |
| | 24 | 1.80 (1.01, 3.19) | 1.16 (0.79, 1.69) | 1.05 (0.72, 1.53) |
| | 48 | 1.33 (0.75, 2.35) | 1.30 (0.89, 1.90) | 1.30 (0.88, 1.92) |
| | 72 | 1.22 (0.69, 2.17) | 1.33 (0.91, 1.94) | 1.02 (0.70, 1.49) |
| **Lead (Pb)** | 0 | 1.00 (Reference) | 1.00 (Reference) | 1.00 (Reference) |
| | 6 | 21.38 (10.59, 43.17) | 23.14 (15.67, 34.16) | 30.04 (21.39, 42.17) |
| | 24 | 2.69 (1.33, 5.42) | 2.47 (1.67, 3.65) | 3.19 (2.27, 4.48) |
| | 48 | 0.99 (0.49, 1.99) | 1.37 (0.93, 2.03) | 1.80 (1.27, 2.55) |

| | | | | |
|---|---|---|---|---|
| | 72 | 1.37 (0.68, 2.77) | 1.32 (0.90, 1.96) | 1.55 (1.10, 2.17) |
| **Antimony (Sb)** | 0 | 1.00 (Reference) | 1.00 (Reference) | 1.00 (Reference) |
| | 6 | 0.92 (0.58, 1.44) | 0.80 (0.51, 1.27) | 4.91 (2.42, 9.96) |
| | 24 | 0.95 (0.61, 1.50) | 0.66 (0.42, 1.04) | 1.28 (0.63, 2.60) |
| | 48 | 1.20 (0.76, 1.89) | 0.68 (0.43, 1.07) | 0.87 (0.42, 1.79) |
| | 72 | 0.88 (0.56, 1.39) | 0.86 (0.53, 1.37) | 0.90 (0.44, 1.83) |
| **Selenium (Se)** | 0 | 1.00 (Reference) | 1.00 (Reference) | 1.00 (Reference) |
| | 6 | 1.21 (0.93, 1.56) | 1.11 (0.83, 1.47) | 1.19 (0.91, 1.57) |
| | 24 | 1.12 (0.86, 1.45) | 0.97 (0.73, 1.28) | 1.15 (0.87, 1.51) |
| | 48 | 1.15 (0.89, 1.49) | 1.00 (0.75, 1.32) | 1.21 (0.91, 1.60) |
| | 72 | 1.02 (0.79, 1.32) | 1.07 (0.80, 1.42) | 1.36 (1.03, 1.78) |
| **Strontium (Sr)** | 0 | 1.00 (Reference) | 1.00 (Reference) | 1.00 (Reference) |
| | 6 | 0.80 (0.55, 1.15) | 0.70 (0.54, 0.90) | 0.47 (0.34, 0.66) |
| | 24 | 1.03 (0.72, 1.49) | 1.06 (0.82, 1.37) | 1.01 (0.72, 1.40) |
| | 48 | 0.85 (0.59, 1.23) | 1.02 (0.78, 1.31) | 0.92 (0.65, 1.30) |
| | 72 | 0.92 (0.64, 1.32) | 1.08 (0.83, 1.39) | 1.03 (0.74, 1.44) |
| **Thallium (Tl)** | 0 | 1.00 (Reference) | 1.00 (Reference) | 1.00 (Reference) |
| | 6 | 0.94 (0.74, 1.21) | 0.91 (0.72, 1.15) | 0.90 (0.64, 1.25) |
| | 24 | 0.95 (0.74, 1.22) | 0.93 (0.74, 1.17) | 0.93 (0.66, 1.30) |
| | 48 | 0.93 (0.73, 1.20) | 0.78 (0.62, 0.98) | 0.93 (0.66, 1.31) |
| | 72 | 0.90 (0.70, 1.16) | 0.91 (0.72, 1.14) | 1.07 (0.77, 1.50) |
| **Uranium (U)** | 0 | 1.00 (Reference) | 1.00 (Reference) | 1.00 (Reference) |
| | 6 | 0.84 (0.40, 1.76) | 1.16 (0.58, 2.32) | 1.03 (0.61, 1.73) |
| | 24 | 1.09 (0.49, 2.44) | 1.07 (0.55, 2.06) | 1.22 (0.71, 2.09) |
| | 48 | 1.06 (0.52, 2.17) | 0.96 (0.48, 1.89) | 0.68 (0.38, 1.20) |
| | 72 | 0.92 (0.44, 1.92) | 1.95 (0.98, 3.86) | 0.82 (0.49, 1.39) |
| **Vanadium (V)** | 0 | 1.00 (Reference) | 1.00 (Reference) | 1.00 (Reference) |
| | 6 | 1.11 (0.88, 1.40) | 1.13 (0.92, 1.39) | 1.42 (1.16, 1.74) |
| | 24 | 1.52 (1.20, 1.92) | 1.61 (1.32, 1.97) | 1.58 (1.29, 1.94) |
| | 48 | 1.39 (1.10, 1.75) | 1.33 (1.09, 1.63) | 1.47 (1.19, 1.82) |
| | 72 | 1.17 (0.93, 1.48) | 1.41 (1.15, 1.73) | 1.31 (1.07, 1.61) |
| **Tungsten (W)** | 0 | 1.00 (Reference) | 1.00 (Reference) | 1.00 (Reference) |
| | 6 | 1.13 (0.60, 2.14) | 0.90 (0.50, 1.61) | 2.08 (1.12, 3.87) |
| | 24 | 1.04 (0.55, 1.97) | 0.86 (0.48, 1.54) | 2.33 (1.25, 4.33) |
| | 48 | 0.72 (0.38, 1.37) | 0.60 (0.34, 1.07) | 1.36 (0.72, 2.58) |
| | 72 | 0.45 (0.24, 0.86) | 0.79 (0.44, 1.41) | 1.31 (0.69, 2.48) |
| **Zinc (Zn)** | 0 | 1.00 (Reference) | 1.00 (Reference) | 1.00 (Reference) |
| | 6 | 20.79 (13.87, 31.17) | 23.15 (16.55, 32.38) | 35.05 (26.04, 47.18) |
| | 24 | 1.48 (0.99, 2.22) | 1.45 (1.04, 2.02) | 2.04 (1.51, 2.74) |

| | | | | |
|---|---|---|---|---|
| | 48 | 1.06 (0.71, 1.59) | 0.91 (0.65, 1.27) | 1.40 (1.03, 1.91) |
| | 72 | 0.97 (0.65, 1.46) | 0.98 (0.70, 1.37) | 1.38 (1.02, 1.85) |

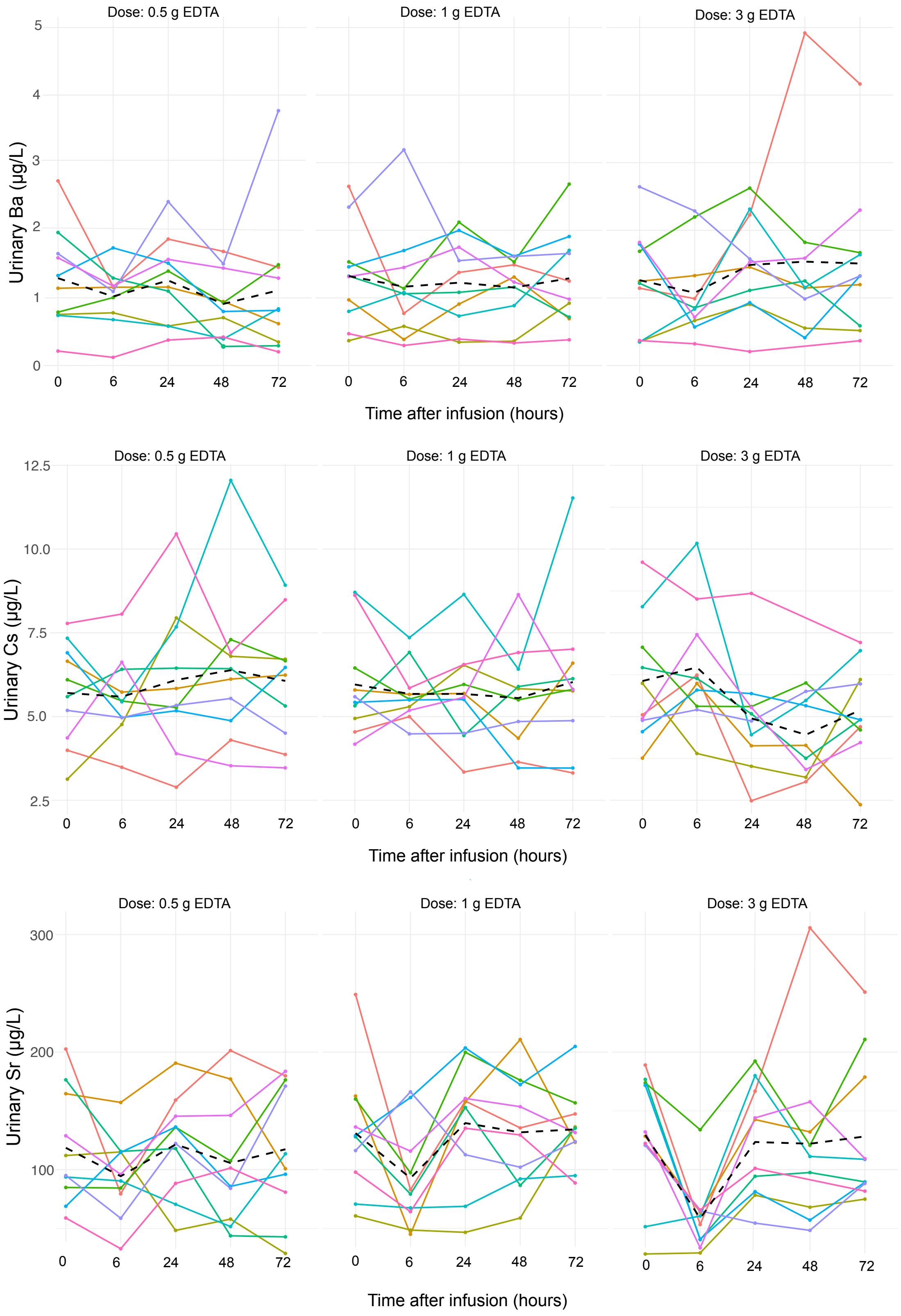
Dose: 0.5 g EDTA
Dose: 1 g EDTA
Dose: 3 g EDTA
Urinary Ba (µg/L)
0
1
2
3
4
5
0
6
24
48
72
Time after infusion (hours)
Dose: 0.5 g EDTA
Dose: 1 g EDTA
Dose: 3 g EDTA
Urinary Cs (µg/L)
2.5
5.0
7.5
10.0
12.5
Time after infusion (hours)
Dose: 0.5 g EDTA
Dose: 1 g EDTA
Dose: 3 g EDTA
Urinary Sr (µg/L)
100
200
300
Time after infusion (hours)

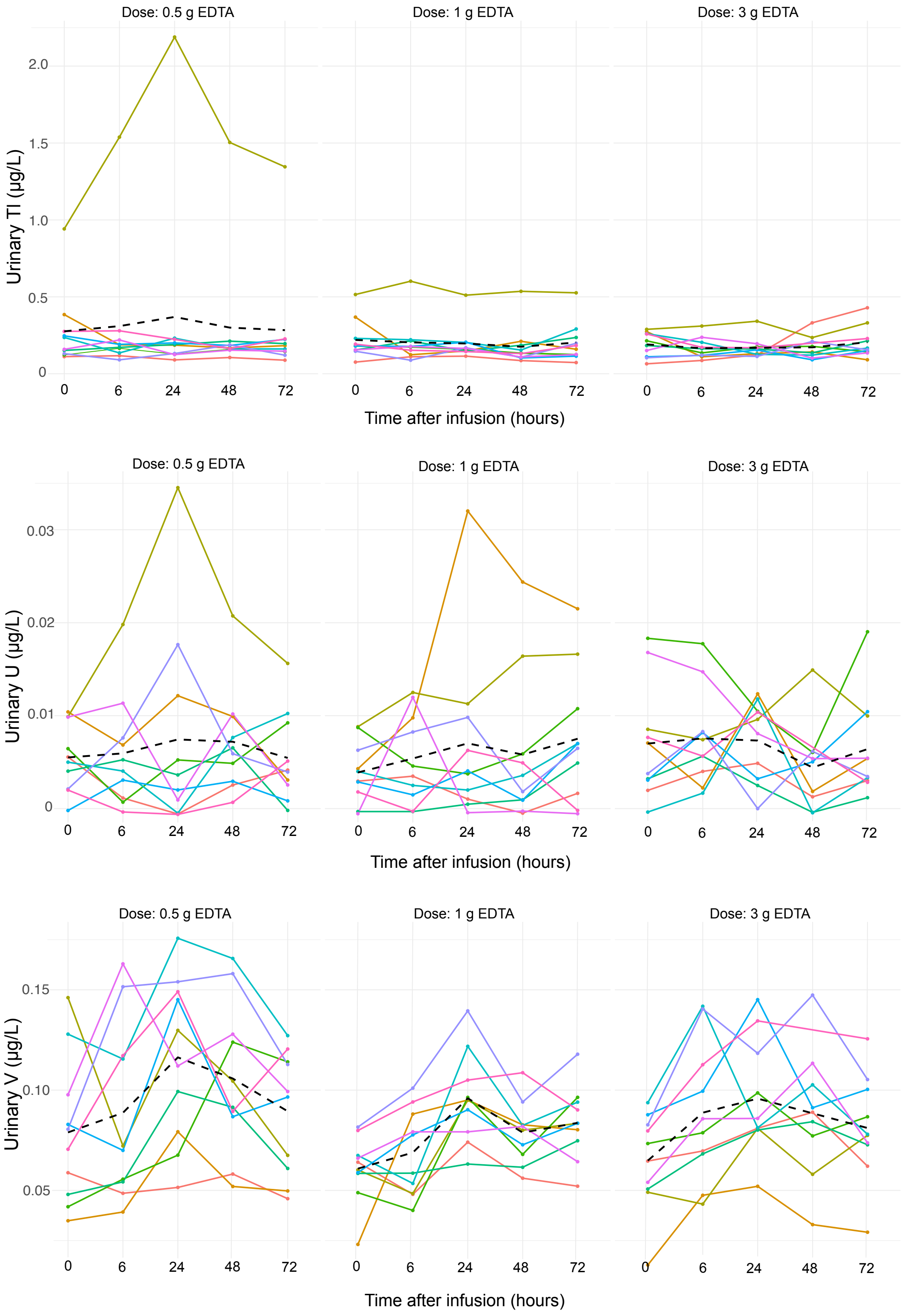
Dose: 0.5 g EDTA
Dose: 1 g EDTA
Dose: 3 g EDTA
Urinary Tl (µg/L)
2.0
1.5
1.0
0.5
0
0
6
24
48
72
Time after infusion (hours)
Dose: 0.5 g EDTA
Dose: 1 g EDTA
Dose: 3 g EDTA
Urinary U (µg/L)
0.03
0.02
0.01
0
0
6
24
48
72
Time after infusion (hours)
Dose: 0.5 g EDTA
Dose: 1 g EDTA
Dose: 3 g EDTA
Urinary V (µg/L)
0.15
0.10
0.05
0
6
24
48
72
Time after infusion (hours)

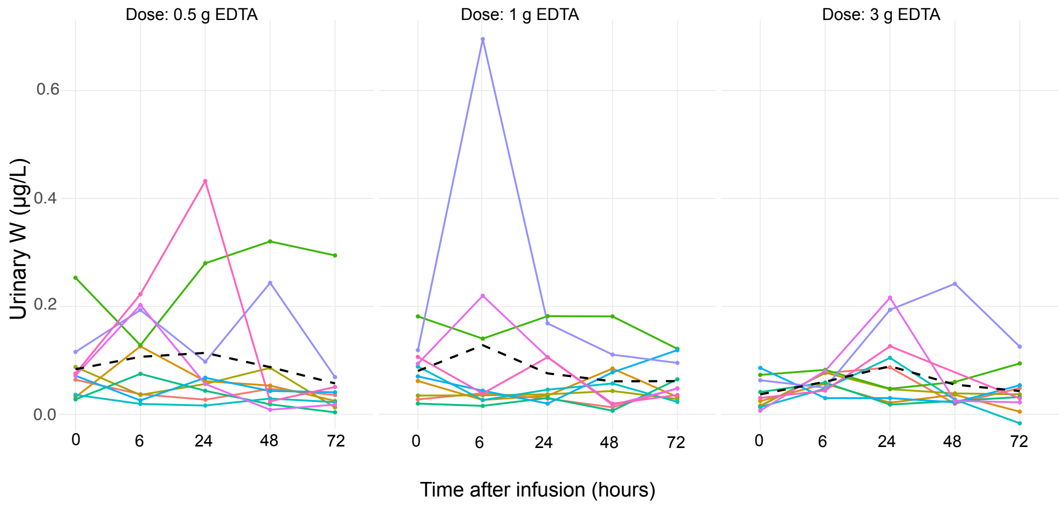


**Figure S1:** Urinary excretion of toxic metals (barium, cesium, strontium, uranium, vanadium, and tungsten) over time (0, 6, 24, 48, and 72 hours) following EDTA infusion at three doses (0.5 g, 1 g, and 3 g). Urinary metal levels were normalized for hydration status using urine specific gravity.
